# Outbreak of *Stenotrophomonas maltophilia* infections in an intensive care unit—Alameda County, California, May–October 2022

**DOI:** 10.1017/ash.2023.351

**Published:** 2023-09-29

**Authors:** Rebeca Elliott, Jeffrey Silvers, Axel Vazquez Deida, Paige Gable, Gillian McAllister, Alyssa Kent, Thomas Ewing, Janet Glowicz, Matthew Arduino, Heather Moulton-Meissner, Mir Noorbakhsh, Patricia Rodrigues, Munira Shemsu, Amit Chitnis, Hilary Metcalf, Barbara Allen, Suada Abdic, Alison Halpin, Kavita Trivedi, Amelia Keaton, Margarita Elsa Villarino

## Abstract

**Background:** *Stenotrophomonas maltophilia* is a gram-negative, biofilm-producing bacterium that is ubiquitous in water environments and often associated with healthcare-associated infections (HAIs). Outbreaks of *S. maltophilia* bloodstream infections are a rare event and raise the suspicion of a common source. We used whole-genome sequencing (WGS) for an investigation of a cluster of *S. maltophilia* HAIs at a single hospital. **Methods:** A patient was defined as an intensive care unit (ICU) patient with fever and *S. maltophilia* isolated from a culture and who was treated for an HAI from May to October 2022. The response to the cluster included an epidemiologic investigation, water infection control risk assessments (WICRA), and environmental sampling. We also conducted WGS to characterize and assess relatedness between clinical and environmental *S. maltophilia* isolates. **Results:** From May 5 to October 1, 2022, we identified 11 HAIs due to *S. maltophilia*: 9 bloodstream infections and 2 ventilator-associated pneumonia cases. The initial epidemiological investigation did not identify common medical products, procedures, or personnel as an exposure source. The WICRA identified several breaches that may have exposed patients to contaminated water from sink backsplashes in the ICU, computerized tomography (CT) rooms, and the emergency department. In the CT rooms, saline bags were sometimes used for multiple patients, as were single-use intravenous contrast solution bottles. No additional cases were identified once infection control breaches were mitigated by installing sink splashguards, disinfecting drains, dedicating sink use for handwashing, and adhering to single-patient use of pharmaceutical products in the CT rooms. Of 46 environmental water samples, 19 were culture-positive for *S. maltophilia*. Isolates available for WGS included 7 clinical isolates (6 blood and 1 respiratory) and 17 environmental isolates. Among the 24 isolates sequenced, 16 unique multilocus sequence types (MLSTs) were identified. The 6 blood isolates sequenced were highly related (ST239, 0–4 high-quality, single-nucleotide variants [hqSNV] over 98.99% core genome), suggesting a common source. Two clusters of related environmental isolates were identified; however, overall MLST and hqSNV analyses suggested no relatedness between clinical and environmental isolates. **Conclusions:** An ICU cluster of *S. maltophilia* bloodstream infections was likely associated with water contamination of room surfaces and use of single-use intravenous products for multiple patients in the setting of a national pharmaceutical product shortage. This investigation highlights the importance of strong surveillance and water infection control, including routine assessment of ancillary areas in which intravenous products are administered and interdisciplinary collaboration to properly mitigate nosocomial transmission.

**Disclosures:** None

